# Transdiagnostic Case Conceptualization of Emotional Problems in Youth with ASD: An Emotion Regulation Approach

**DOI:** 10.1111/cpsp.12084

**Published:** 2014-12-15

**Authors:** Jonathan A Weiss

**Affiliations:** York University

**Keywords:** autism spectrum disorder, cognitive behavior therapy, emotion regulation, transdiagnostic

## Abstract

Youth with autism spectrum disorder often struggle to cope with co-occurring anxiety, depression, or anger, and having both internalizing and externalizing symptoms is a common clinical presentation. A number of authors have designed cognitive-behavioral interventions to address transdiagnostic factors related to multiple emotional problems, although none have applied this focus to youth with ASD. The current review article describes how a transdiagnostic emotion regulation framework may inform cognitive-behavioral interventions for youth with ASD, which until now have focused almost exclusively on anxiety. Research is needed to empirically test how a transdiagnostic intervention can address the processes of emotion regulation and assist youth with ASD to cope with their emotional disorders.

## Introduction

Emotional and behavioral difficulties are a serious set of associated issues for individuals with autism spectrum disorder (ASD) who by definition of their diagnosis of ASD already struggle with pervasive impairments in sociocommunicative functioning and restricted or repetitive behaviors or interests. For the purposes of this review, I view emotional and behavioral problems as symptoms that lead to significant impairment above the individual's baseline level of impairment related to his or her symptoms of ASD (i.e., above the impairment associated with the core social, communication, and repetitive features of ASD; as in Leyfer et al., 2006). Distinguishing core symptoms of ASD from comorbid internalizing and externalizing symptoms is particularly challenging, and a number of authors suggest that while some symptoms may reflect distinct disorders with unique etiologies, other symptoms are likely “co-occurring” issues, related to the etiology of the ASD or associated with symptoms of ASD (see also the commentaries by Folstein & Carbajal, 2012; Kerns & Kendall, 2012; Ollendick & White, 2012; Scahill, 2012; Wood & Gadow, 2010).

Rates of emotional and behavioral problems are high in youth with ASD, either when viewed as comorbid or as co-occurring. Using a population-based cohort, Totsika, Hastings, Emerson, Berridge, and Lancaster (2011) report that 59% of 5-year-olds with ASD and no intellectual disability have clinically significant levels of hyperactivity, 46% have clinically significant conduct problems, and 38% have clinically significant emotional problems. Across 5- to 16-year-olds with ASD and no intellectual disability, 85% are noted to have clinically significant levels of hyperactivity, 74% with clinically significant levels of emotional problems, and 64% with clinically significant conduct problems (Totsika, Hastings, Emerson, Lancaster, & Berridge, 2011; with similar rates for youth with ASD and intellectual disability). Using an adaptive psychiatric interview that distinguished ASD symptoms from other Axis I psychiatric symptoms, Simonoff et al. (2008) estimate that 70% of 12- to 16-year-olds with ASD meet criteria for at least one comorbid condition, with anxiety disorders being the most common set of disorders, at 42%. Similarly, Leyfer et al. (2006) developed the Autism Comorbidity Interview to distinguish between ASD-related social avoidance from social anxiety disorder and ASD-related agitation from generalized anxiety and found that 72% of their sample met criteria for at least one *DSM-IV* Axis I condition.^1^ These emotional and behavioral problems place a high burden on the individual, his or her family caregivers, and service providers, and detract tremendously from individual and family quality of life.

There is also substantial overlap of internalizing and externalizing symptoms in people with ASD. Approximately 40–50% of youth with ASD will meet criteria for two or more psychiatric disorders when using psychiatric interviews adapted to take into account ASD symptoms (Leyfer et al., 2006; Simonoff et al., 2008). Individuals with ASD who have high levels of depression or anxiety also show increased levels of noncompliance, aggressive behavior, and irritability (Kim, Szatmari, Bryson, Streiner, & Wilson, 2000; Matson & Nebel-Schwalm, 2007; Tantum, 2000), and difficulties with anger, depression, and anxiety are found to be correlated, both when using self-report (Quek, Sofronoff, Sheffield, White, & Kelly, 2012) and parent report data (Hurtig et al., 2009). Numerous studies of children with ASD highlight correlations within and between internalizing and externalizing symptoms (Gadow, DeVincent, & Pomeroy, 2006; Lecavalier, Gadow, DeVincent, & Edwards, 2008; Leyfer et al., 2006; Weisbrot, Gadow, DeVincent, & Pomeroy, 2005). This overlap is not surprising, given that even in the general population, both anxious and depressed individuals are noted to have associated difficulties with sadness, anger, and anxiety (Rohde, 2012). Such data have led authors to suggest that there may be a shared mechanism underlying problems with emotions (Trosper, Buzzella, Bennett, & Ehrenreich, 2009). They suggest that the various expressions of psychopathology are the manifestation of a broader syndrome, with “shared psychosocial and biological diatheses” (Barlow, Allen, & Choate, 2004, p. 210).

The fact that individuals with various psychiatric diagnoses may share common underlying problems has led many to suggest that there are limitations to a disorder-specific approach to treating emotional problems in the general population (Dudley, Kuyken, & Padesky, 2011). These issues may well extend to treating emotional problems in people with ASD, given the aforementioned rates. On a practical level, the current disorder-specific approach to treatment limits our ability to address multiple comorbidities in a real-world context. Clinicians are often asked to provide treatment to individuals with ASD who do not demonstrate symptoms that map efficiently onto diagnostic categories, yet who continue to struggle with stressors. A stress generation dynamic has been described to explain the nature of anxiety disorders within the context of ASD and can be readily applied to explain other emotional problems, such as depression or anger (Wood & Gadow, 2010). In their model, Wood and Gadow (2010) suggest that ASD-related stressors (of social confusion; peer rejections and victimization related to ASD symptoms; prevention or punishment of preferred behaviors or interests; and frequent aversive sensory experiences) lead to increased overall negative affectivity, anxiety disorders (depending on the experiences, social phobia, obsessive-compulsive disorder, generalized anxiety, or separation anxiety), or depression. In turn, such negative affectivity contributes to more ASD symptoms, conduct problems, and personal distress, which lead to even further ASD-related stressors, completing a negative cycle. Wood and Gadow's (2010) model is not limited to one form of anxiety disorder or even anxiety disorders more broadly. By explaining the cycle of a broad range of stressors and emotion problems, the model highlights how transdiagnostic mechanisms may be important contributors to psychopathology in youth with ASD.

## The Need for a Transdiagnostic Cognitive-Behavioral Approach

While there does exist an important and growing literature on the efficacy of cognitive behavior therapy (CBT) for anxiety disorders in youth with ASD (McNally Keehn, Lincoln, Brown, & Chavira, 2013; Reaven, Blakeley-Smith, Culhane-Shelburne, & Hepburn, 2012; Sofronoff, Attwood, & Hinton, 2005; White, Ollendick, Scahill, Oswald, & Albano, 2009; Wood et al., 2009), the majority of this treatment research does not account for youth who specifically have co-occurring depression or anger problems, and none have examined commensurate changes in these other important emotional domains. Further, there is far less evidence demonstrating ways of treating conduct problems, oppositionality, attentional problems, or depression in individuals with ASD, although some promising initial pilot work is emerging in these areas (Scarpa & Reyes, 2011; Singh et al., 2011; Sofronoff, Attwood, Hinton, & Levin, 2007; Spek, van Ham, & Nyklíček, 2013). Having co-occurring disorders is known to be related to poor prognosis in and following therapy for typically developing individuals (Rohde, 2012), which may also explain why a significant proportion of youth with ASD in treatment for an anxiety disorder continue to exhibit problems with anxiety following the disorder-specific intervention (McNally Keehn et al., 2013; Reaven et al., 2012; Wood et al., 2009). Relying solely on a diagnosis-driven approach may make it difficult to find interventions to address the underlying factors that might lead to any combination of anxiety, depression, or anger-related problems, which, if addressed, could facilitate change across several presenting problems. At the same time, Dudley et al. (2011) note that although disorder-specific models may emphasize differences in treatment applicability, there are considerable similarities among evidence-based manuals, which may in fact be helpful to address multiple emotional problems. This exists even within the very limited CBT literature for children with ASD, with two randomized controlled trials (RCTs) using almost exactly the same “tool kit” for anger and anxiety (Sofronoff et al., 2005, 2007).

A number of cognitive-behavioral interventions exist targeting transdiagnostic factors that are common across presenting problems, in individuals without ASD (Boisseau, Farchione, Fairholme, Ellard, & Barlow, 2010; Ehrenreich, Goldstein, Wright, & Barlow, 2009). While a systematic review of the evidence for the effectiveness of transdiagnostic approaches for youth and adults is beyond the scope of this review, there are a number of findings that suggest that a transdiagnostic conceptualization can inform treatment planning. For instance, a recent RCT in adults with anxiety disorders (without ASD) using group transdiagnostic CBT, designed to address multiple types of anxiety disorders (e.g., used in a uniform way to target social anxiety, generalized anxiety disorder, and specific phobia), was found to be as efficacious as disorder-specific CBT for these conditions (Norton & Barrera, 2012) and more efficacious than a group relaxation training program (Norton, 2012). This group transdiagnostic treatment also appears to result in greater improvements in comorbid mood disorders compared to diagnostic-specific CBT from previous studies (rates of 67% improvement in the transdiagnostic group versus approximately 49% in benchmarking studies; Norton et al., 2013). Other research groups have demonstrated that transdiagnostic CBT for adults with anxiety and depression is efficacious compared to waitlist controls, using RCT designs (Farchione et al., 2012; Johnston, Titov, Andrews, Spence, & Dear, 2011; Titov et al., 2013), and may assist in long-term relapse prevention compared to treatment as usual for adults who received inpatient CBT (Ebert, Tarnowski, Gollwitzer, Sieland, & Berking, 2013). The research on transdiagnostic CBT for children or youth with anxiety and depression is less developed, leaving many to call for more research using RCT designs (Kendall, Settipani, & Cummings, 2012; Rohde, 2012). To date, most research on transdiagnostic CBT in youth is limited to case studies (e.g., Ehrenreich-May & Bilek, 2012) or open trials (Bilek & Ehrenreich-May, 2012), although ongoing RCT studies appear promising (Ehrenreich-May, Queen, Bilek, Remmes, & Marciel, 2013).

Emotion regulation is often a core focus of these transdiagnostic models (McLaughlin, Hatzenbuehler, Mennin, & Nolen-Hoeksema, 2011), which has been implicated in over half of *DSM-IV* Axis I and all of the Axis II disorders (Chambers, Gullone, & Allen, 2009). For example, the Unified Protocol for the Treatment of Emotional Disorders is a transdiagnostic cognitive-behavioral intervention for adults with anxiety or depression (UP; Barlow et al., 2011; Farchione et al., 2012) that focuses on the internal emotional experience and emotion regulation, employing commonly used strategies of psychoeducation, reappraisal, and exposure (for a discussion of its development and how it differs from traditional CBT, see Ellard, Fairholme, Boisseau, Farchione, & Barlow, 2010). The UP was recently adapted for children and adolescents (UP-Y; Ehrenreich et al., 2009; Trosper et al., 2009), with a focus on treating anxiety, depression, and anger. The eight-module intervention consists of five mandatory modules (emotion education, emotion awareness, cognitive flexibility, exposure to distressing emotions, and relapse prevention) and three optional modules (motivational enhancement, crisis management, and parenting skills). Youth remain in the module until they demonstrate mastery over the material (up to 21 sessions in total). Preliminary research with adolescents demonstrates significant reduction in anxiety and depressive symptomatology, and an increase in parent-reported child emotion regulation (Bilek & Ehrenreich-May, 2012). Ehrenreich-May and colleagues’ pioneering application of an emotion regulation framework (Gross & Thompson, 2007) stands to inform how we address emotional problems in youth with ASD, who struggle in many respects to a greater degree with emotion regulation than typically developing peers. The following article outlines a developmental contextual emotion regulation framework to assist in the treatment planning for youth with ASD who struggle with multiple emotional problems.

## Emotion Regulation Formulation

Emotion regulation is defined as the process of effectively managing emotions in response to environmental demands and has been implicated in conceptualizations of emotional disorders in children and adults (see Aldao, Nolen-Hoeksema, & Schweizer, 2010, for a comprehensive review). Emotion regulation strategies are typically initiated, either consciously (effortful and controlled) or unconsciously (effortless and automatic), in response to emotion-eliciting stimuli, with the aim of altering the degree or type of a person's emotional experience or the stimuli (Aldao & Nolen-Hoeksema, 2010). Successful emotion regulation, through the use of adaptive and the disuse of maladaptive strategies, has been implicated as an instrumental act for positive outcomes in children (Garnefski, Rieffe, Jellesma, Terwogt, & Kraaij, 2006). Whether an emotion regulation strategy is adaptive is determined by its outcome, as it refers to processes that are successful in achieving the appropriate affect and do not have negative long-term costs (Campbell-Sills & Barlow, 2007). This last point is important, as it sets the stage for determining whether a particular emotion regulation behavior should be promoted to reduce distress or seen as a problematic behavior. For instance, a child may have a specific ritual that he or she likes to perform to reduce test-taking anxiety (e.g., making sure his or her desk is organized correctly and counting to five quietly before starting). When put into practice, this ritual may be adaptive if there are no negative consequences to it (i.e., it does not lead to social, personal, or other negative outcomes) and if it does not interfere with test taking. Instead, it helps the child control his or her stress and focus on the task at hand. This same behavior may be considered maladaptive if adherence to it qualifies as an obsession and interference with it leads to behavioral issues or problems with the ability to take the test. It may also be considered maladaptive if it leads to additional negative outcomes, such as social isolation.

A number of recent review articles suggest that problems with emotion regulation may explain many of the emotional and behavioral problems in individuals with ASD. Mazefsky et al. (2013) outline the ways in which emotion regulation impairments may be related to ASD. Given the high rates of psychiatric comorbidities, it may be that a comorbid psychiatric condition impacts emotion regulation skills in youth with ASD. Conversely, it may be that individuals with ASD have core deficits in emotion regulation, which then account for emotional and behavioral problems in this population, similar to the research on transdiagnostic factors associated with multiple psychiatric comorbidities in the typically developing population (e.g., McLaughlin et al., 2011). It may also be that the same emotion regulation impairments that are associated with the onset of anxiety and mood disorders are related to the presentation of symptoms of ASD, with shared underlying clinical and biological mechanisms. For instance, emotion regulation occurs in the prefrontal and ventromedial prefrontal cortex, including the amygdala (Kross, Davidson, Weber, & Ochsner, 2009), and these same areas have been implicated in the social impairments found in individuals with ASD (Gallager & Frith, 2003; see Mazefsky et al., 2013, for a detailed review of the neurological basis of emotion regulation deficits in individuals with ASD).

Ultimately, Mazefsky and colleagues suggest that emotion regulation deficits in individuals with ASD are related to interactions among many elements, including the presence of neural circuitry that explains both psychiatric issues and ASD symptoms (e.g., abnormal amygdala/prefrontal cortex function and connectivity), of psychiatric conditions, of greater baseline levels of negative affectivity and hyperarousal, and of ASD-related behavioral (e.g., lack of social motivation, awareness of emotion) and cognitive features (e.g., perseveration, rigidity, poor problem solving, and information processing). Given the many different potential interactions, it is likely that multiple profiles of emotion regulation characterize individuals with ASD (Mazefsky, Pelphrey, & Dahl, 2012), and a framework with which to organize emotion regulation is important to understand individual differences and determine specific ways of intervening (Mazefsky & White, 2014; Sofronoff, Beaumont, & Weiss, 2014).

**Figure 1:**
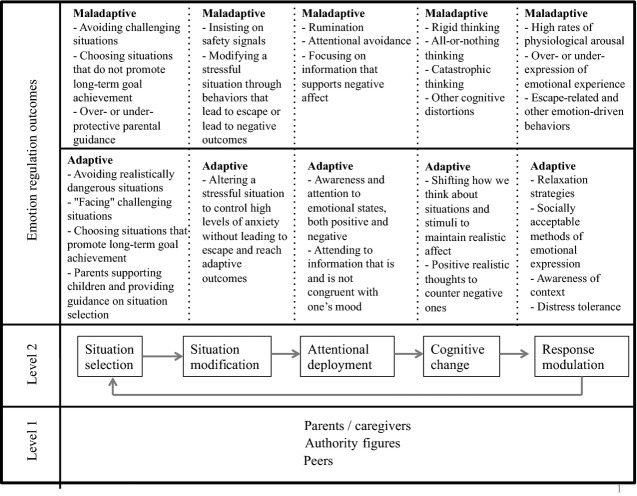
Transdiagnostic emotion regulation model. The five processes of Gross and Thompson's (2007) modal model of emotion regulation, with each one leading to the next, and response modulation affecting one's situation selection or other ER domains (Level 2). Successful emotion regulation, at any one domain, is considered an individual–relational interaction with parents/caregivers, peers, and other authority figures (e.g., teachers; Level 1). Each process may be “adaptive” or “maladaptive,” depending on any given strategy's short- and long-term outcomes.

Gross and Thompson (2007) describe five temporally linked domains of a person–situation interaction where emotions can be regulated: situation selection, situation modification, attentional deployment, cognitive change, and response modulation. By employing a transdiagnostic case conceptualization that examines each component, clinicians can foster a child's internal skills to help him or her regulate emotions when confronted with challenges. For instance, these five components serve as the framework of Ehrenreich-May's UP-Y program, informing intervention design to address emotion regulation problems in youth without ASD. Understanding how youth with ASD demonstrate maladaptive and adaptive emotion regulation processes can greatly assist treatment planning. Figure1 presents common adaptive and maladaptive aspects of each component, and how it can be examined with an ASD lens (described below).

### Situation Selection

Situation selection (Figure1) involves the “purposeful approach toward situations believed to result in pleasant emotions, and avoidance of situations believed to result in undesirable emotions” (Trosper et al., 2009, p. 238). Staying away from a truly dangerous situation is an example of adaptive situation selection. Successful situation selection requires an understanding of context and an ability to estimate probable outcomes. While many individuals with ASD may have no trouble understanding familiar situations that have concrete and predictable (and particularly nonsocial) rules, novel situations that are social and unstructured decrease the likelihood of an adaptive response (Baron-Cohen, Ring, et al., 1999; Baron-Cohen, Wheelwright, Stone, & Rutherford, 1999; Lawson, Baron-Cohen, & Wheelwright, 2004; Minshew, Meyer, & Goldstein, 2002). Such situations require tolerance for uncertainty, learning new rules, flexibility, understanding of context, and theory of mind, all demands that are often challenging for individuals with ASD (Gomot & Wicker, 2012). The insistence on routine and ritual exhibited by many youth with ASD, which has been associated with emotional problems (Gotham et al., 2013), may lead to an increased likelihood of choosing a limited number of pleasurable and familiar situations. Adaptive situation selection also requires that children have opportunities to choose to experience a variety of situations, and many parents report issues around a lack of goodness-of-fit between a child with ASD's needs and the available environmental supports, thereby limiting situational options (Hodgetts, Nicolas, & Zwaigenbaum, 2013; Orsmond, Krauss, & Seltzer, 2004).

Maladaptive situation selection typically refers to problematic avoidance of situations. Avoidance has long been implicated within cognitive and learning models of emotional disorders, particularly as a mechanism that maintains the association between the stimulus and emotional response, through negative reinforcement. It also prevents the process of extinction and habituation from occurring (which would weaken the conditioning). Avoidance is a common element of anxiety disorders (Aldao et al., 2010; Rapee, 2002), and depression, where individuals withdraw from pleasurable activities and social situations (Mash & Wolfe, 2002). It further compounds anger responses as well, when there is a likelihood that the situation will arise in the near future and is believed to be threatening. When confronted with the possibility of negative emotion-eliciting situations, children with ASD have been shown to resort to avoidance more than typically developing peers (Jahromi, Meek, & Ober-Reynolds, 2012) and may act in ways to avoid being exposed to the situation, never building the skills or confidence related to it. Youth with ASD are at increased risk of avoiding novel situations as a result of insistence on routine and ritual (Gotham et al., 2013), further failing to experience opportunities for increased competence and confidence. Similarly, they are at greater risk of avoiding or withdrawing from social situations as a result of impairments in sociocommunicative skill (Dawson & Lewy, 1989; Jawaid et al., 2012), losing important opportunities for social skills growth and social engagement (Orsmond et al., 2004). Facing emotion-eliciting situations through graduated exposure is a foundational element of cognitive behavior therapy for children with and without ASD (Munoz-Solomando, Kendall, & Whittington, 2008; Reaven et al., 2012; Wood et al., 2009). The use of social stories is another strategy often employed to address weaknesses in dealing with novel situations, understanding, and predictive skill (Ivey, Heflin, & Alberto, 2004). Further, the application of priming as a means of exposure to assist in transitions or to new situations (Schreibman, Whalen, & Stahmer, 2000), and programming for the generalization of skills to new situations (Kleeberger & Mirenda, 2010), is viewed as an evidence-based strategy in applied behavior analysis for children with ASD.

### Situation Modification

Situation modification (Figure1) involves altering situations to address emotional responses. Successful problem solving, defined as the conscious act of modifying the emotion-eliciting situation or its consequences (Snyder, 2010; Spering, Wagener, & Funke, 2005), represents adaptive situation modification. Individuals with ASD are known to have difficulties with social and “real-world” problem solving (Channon, Charman, Heap, Crawford, & Rios, 2001), which can lead them to act in inappropriate ways or serve to make the problem situation worse. For example, if faced with a stressful situation at school, an effective solution may be to speak to the teacher, and by doing so, could serve to modify the situation in an appropriate way. An ineffective solution might be to lose control over one's anger and act aggressively in class. Both options “solve” the problem by modifying the environment, yet one is clearly more adaptive than the other. Helping children with ASD to problem-solve effectively is thought to be an important part of improving their adaptive emotion regulation (Magyar & Pandolfi, 2012).

A more subtle maladaptive modification would be to *overly* rely on “safety signals” to indicate that an aversive stimulus will not occur in a situation (Campbell-Sills & Barlow, 2007). Safety signals “can be inanimate objects (e.g., a certain room in the home), behaviors (e.g., sitting at a desk close to an exit), or other people (e.g., a parent)” (Trosper et al., 2009, p. 238). An overreliance on safety signals teaches an individual to depend on the external world for control and safety and leads to a dependence on the external signal for temporary emotional relief, instead of learning that he or she can manage successfully in the emotion-eliciting situation. The behavioral rigidities found in ASD may serve as safety signal behaviors, by perseverating on those cues that maintain emotional relief (Howlin, 2004). For instance, a child who must keep his or her desk in a specific way to avoid feeling uncomfortable in class is relying on that rule for regulation. When children are able to identify and distance themselves from an overreliance on safety signals, they develop a positive internal locus of control, which is related to reduced anxiety, depression, and anger (Clark, Olympia, Jensen, Heathfield, & Jenson, 2004). Finally, youth with ASD who exhibit an intense adherence to routine and ritual may demonstrate maladaptive situation modification by attempting to ensure that their environments remain exactly the same, a violation of which can result in a host of emotionally driven responses (Barnhill, 2007; Dominick, Davis, Lainhart, Tager-Flusberg, & Folstein, 2007). It is also clear that behavioral rigidities in ASD serve a host of functions in addition to those that connote safety, such as sensory reinforcement or producing adult attention, and that understanding this function is instrumental for developing effective behavioral interventions (Kennedy, Meyer, Knowles, & Shukla, 2000).

When we do not have an opportunity to choose or modify our situations, we continue to regulate our emotions via our attentional deployment (Figure1), which involves how we choose to focus or distance our attention on the situation. Three aspects of attentional deployment are considered here: (a) emotional awareness, (b) mood-congruent attentional bias, and (c) rumination. Emotional awareness is defined “as an attentional process that serves to monitor and differentiate emotions, locate their antecedents, but ignore the physical arousal that is part of the emotion experience” (Rieffe et al., 2011, p. 656) and has been implicated as a prerequisite process to successful attentional deployment. Children with ASD have difficulties with emotional awareness of themselves and others (Tanaka et al., 2012; Williams & Happé, 2010), including difficulties assessing and labeling their own emotions (i.e., alexithymia; Fitzgerald & Bellgrove, 2006; Hill, Berthoz, & Frith, 2004; Szatmari et al., 2008). Poor emotion awareness has been linked to internalizing symptoms in children with and without ASD (Rieffe et al., 2011) and is a barrier to constructive help seeking and problem solving (Zeman, Shipman, & Suveg, 2002). Helping children to learn about emotional signals, and how emotions are related to their thoughts and actions, can improve the ability to control emotions (Friedberg & McClure, 2002; Scarpa & Reyes, 2011). In fact, teaching about emotions is a common aspect of well-known behavioral early intervention programs for children with ASD (Smith, 2006).

When individuals struggle with emotions, they often show an attention bias on information that is related to the problem emotion, confirming its validity by being more likely to notice situational signals that are in line with the emotion and less able to see the signals that are not. Regardless of whether the bias is toward threat-related cues (common in anxiety and anger; Bögels & Zigterman, 2000; Koster, Verschuere, Crombez, & Van Damme, 2005; Mineka, Rafaeli, & Yovel, 2003; Williams, Mathews, & MacLeod, 1996; Yovel & Mineka, 2005) or hopeless and rejection-related cues (common in depression; Williams et al., 1996; Yovel & Mineka, 2005), children benefit from seeing the positive and neutral elements of their situations (Groden, Kantor, Woodard, & Lipsitt, 2011). Children with ASD may be at greater risk to focus on negative or irrelevant cues (Embregts & van Nieuwenhuijzen, 2009), and although they are often detail oriented (O'Riordan, Plaisted, Driver, & Baron-Cohen, 2001), they may miss the most contextual and central information required to counter their emotional states (Farrugia & Hudson, 2006). According to parent report, children with ASD are often less able to focus and shift their attention on emotionally relevant stimuli compared to peers, indicating that their attentional biases may be highly rigid (Bailey, Hatton, Mesibov, Ament, & Skinner, 2000; Hepburn & Stone, 2006; Janes, 2001; Konstantareas & Stewart, 2006; Landry & Bryson, 2004). Rumination is a related maladaptive attentional strategy, defined as “a pattern of responding to distress in which an individual passively and perseveratively thinks about his or her upsetting symptoms and the causes and consequences of those symptoms, while failing to initiate the active problem solving that might alter the cause of that distress” (McLaughlin & Nolen-Hoeksema, 2011, p. 186). Rumination includes excessive worry and brooding, which can lead to emotional distress in children and youth with ASD (Rieffe et al., 2011). As children with ASD are predisposed to perseverate, they are at risk of rumination, and interventions are required to improve their abilities to shift their focus toward positive topics when needed.

It is important to note that the mechanisms underlying rumination in children with and without ASD may differ. Problems with attention in individuals with ASD extend beyond a problematic focus on emotionally laden stimuli or situations, or with coping with distress, and may instead be caused by broader deficits in executive functions, including response inhibition, working memory, cognitive flexibility (set-shifting), planning, and fluency (Ozonoff & Strayer, 1997; Pennington & Ozonoff, 1996). It is well known that children with ASD have difficulties shifting attention from one activity, rule, or interest, even when the focus is not distressing and there is no negative affect (Ozonoff, Strayer, McMahon, & Filloux, 1994), suggesting that perseveration is related to overall difficulties with cognitive control. This speaks to broader impairments in attention as potential risk factors and causes for rumination in individuals with ASD, rather than the emotional experience being the cause. (Mazefsky, Oswald, et al., 2012; Mazefsky, Pelphrey, et al., 2012) suggest that motivationally significant topics, such as intense interests, may lead to dampened cognitive flexibility and to impaired ability to disengage attention and that this reduced ability can lead to emotion regulation problems. This broader cause of rumination in individuals with ASD highlights the need for interventions to address cognitive flexibility and attentional difficulties beyond emotion regulation to assist individuals with ASD in emotion regulation tasks. Such attentional deployment is a hallmark of mindfulness-based interventions, which aim to bring attention to emotions in a nonjudgmental way, promoting acceptance and a move away from both suppression and rumination (de Bruin, Zijlstra, & Bögels, 2014). In adults with ASD, mindfulness-based cognitive therapy has been successfully used to reduce rumination, anxiety, and depression (Spek et al., 2013). In young children with ASD, particular interventions have focused on improving joint attention between mother and child, and this improvement in joint attention has led to improvements in emotion regulation (Gulsrud, Jahromi, & Kasari, 2010).

Even if we cannot control the situation or our attention to it, our emotional reactions can be modified through our thoughts about the situation and our capacity to cope with it, known as cognitive change (Figure1; Gross & Thompson, 2007). Reappraisal typically involves using evidence to alter the valence or intensity of the experience (Trosper et al., 2009). Developing reasonable interpretations of a distressing situation (i.e., reappraisal), rather than directly attempting to change it, can lessen its emotional impact (Aldao et al., 2010). Reappraisal also occurs through the interpretations of the meanings we place on the emotions themselves (Gross & Thompson, 2007). Like all emotion regulation strategies, reappraisal is a developmental process, which only fully emerges at approximately age 10 years (as cited in Chambers et al., 2009; Ochsner & Gross, 2005). Prior to this age, more concrete levels of reappraisal continue to be effective, such as using positive statements to offset negative ones, and it is these that can most help youth with ASD. Children with ASD are at great risk of employing maladaptive cognitive change strategies, including “all-or-nothing” and catastrophic thinking, which can impact their interpretations of emotion-eliciting situations (Attwood, 2004; Fujii et al., 2013). They have been shown to be capable of developing cognitive change strategies, including coping statements and thinking in more neutral or positive ways about distressing situations, to counteract these cognitive distortions (Beaumont & Sofronoff, 2008; Reaven et al., 2012; Sofronoff et al., 2005).

### Response Modulation

Response modulation (Figure1) involves the physiological and behavioral ways of regulating emotion after it is felt. For example, relaxation strategies are meant to lessen physiological arousal, resulting in a calmer emotional state (Weersing, Rozenman, Maher-Bridge, & Campo, 2012). Children with ASD are less able to sooth and are more sensitive to negative stimuli than matched peers (Janes, 2001; Konstantareas & Stewart, 2006; Landry &Bryson, 2004). They may also exhibit an overall higher rate of physiological arousal (Bal et al., 2010; Hirstein, Iversen, & Ramachandran, 2001; Kylliäinen & Hietanen, 2006). If used appropriately, relaxation strategies, such as progressive muscle relaxation, can help children with ASD to experience a negative emotion at a suitable level, while staying in the situation to benefit from exposure (Mullins & Christian, 2001; Sofronoff et al., 2005, 2007).

Response modulation also involves how emotions are behaviorally expressed. Trosper et al. (2009) note that emotional expression lies on a continuum from inhibition, to appropriate expression, to exaggeration of emotional response. Children with emotional disorders are known to show both an under- and overcontrol of emotional expression, by inhibiting emotional expression and by expressing it in nonconstructive, excessive ways (Zeman et al., 2002). The appropriate expression of emotion is influenced by how it is modeled by others and the larger social context (Gross & Thompson, 2007; Zeman & Shipman, 1998), and youth with ASD are often susceptible to missing such cues (Loveland, Pearson, Tunali-Kotoski, Ortegon, & Gibbs, 2001). As such, an important part of intervention is to help youth to understand the social situation and how best to express their feelings *(*Friedberg & McClure, 2002; Williams, Gray, & Tonge, 2012). Teaching behavioral self-management strategies to children with ASD is a common aspect of applied behavior analysis and can include differentially reinforcing prosocial ways of expressing emotions (Lee, Simpson, & Shogren, 2007).

As avoidance is to situation selection, escape-related behaviors are to response modulation, implemented once a person experiences a distressing emotion in an effort to reduce the intensity. When objectively dangerous situations exist, escape is an important adaptive strategy of responding to an emotional signal. However, individuals with emotional difficulties often aim to escape situations that they interpret as threatening or undesirable, and by doing so, serve to maintain the contingency linking the negative emotion to the situation (Trosper et al., 2009). As such, response modulation is thought to influence one's situation selection (Gross & Thompson, 2007). Such emotion-driven behaviors take many forms, including withdrawal, aggression, or flight (Barlow et al., 2004), and children with ASD are well known to react to negative emotions with behaviors that serve escape functions (Jahromi et al., 2012). They also struggle more to tolerate experiencing negative affect compared to peers (Goldstein & Schwebach, 2004; Loveland & Tunali-Kotoski, 1997). There is a robust literature in applied behavior analysis indicating how differential reinforcement can address behaviors that serve escape functions (e.g., Hagopian, Wilson, & Wilder, 2001; Harper, Iwata, & Camp, 2013). Helping children with ASD endure negative affect in an acceptable way, without attempting to escape, is a critical component of helping them manage their emotions (Singh et al., 2011).

## A Developmental Contextual Framework of Emotion Regulation

Emotion regulation is described as an individual-context transactional process (Figure1, Level 1; Gross & Thompson, 2007) embedded within an ecological framework of multiple systems of influence (Bronfenbrenner, 1986, 1992). That is, the development and continued use of emotion regulation require an alignment among a child's internal capabilities and the provision of ecological resources found in the family, school, and larger community (often described as “developmental assets”; Benson, 2006; Lerner, 2005). The comprehensive model of positive youth development (or thriving) articulated by Lerner and colleagues provides an ideal example of how an ongoing process of emotion regulation requires particular child characteristics and external supports (Geldhof, Weiner, Agans, Mueller, & Lerner, 2014). We are influenced by, and influence, our ecological assets, which include “the presence, quantity, and accessibility of human, material, and social resources in the environment” (Lerner, 2005, p. 40).

There are indeed key individuals who are instrumental in promoting successful emotion regulation in youth with and without ASD. Parents play a critical role supporting all children in selecting appropriate situations, scaffolding skills and the development of confidence, and modeling appropriate regulation strategies (Gross & Thompson, 2007). Scaffolding is defined as a process where a more experienced individual (e.g., parent) provides assistance to the child in a way that helps him or her develop within a zone of proximal development (Harrop & Green, 2012; Vygotsky, 1978) and involves three overarching components: contingency, fading, and transfer of responsibility (van de Pol, Volman, & Beishuizen, 2010). A number of parent strategies, which appear to target specific emotion regulation domains as discussed above, can be used to scaffold (Reiss, unpublished manuscript): distraction (altering attentional deployment), reassurance (as a safety signal; situation modification), encouragement/praise (response modulation), guidance/problem solving (situation modification), following/elaborating (elaborating on a child's distress), or direction/control (verbally or physically directing a child's attentional deployment or situation selection; Calkins & Johnson, 1998; Eisenberg & Fabes, 1994; Grolnick, Kurowski, & McMenamy, 1998; Rubin, Hastings, Chen, Stewart, & McNichol, 1998). In the context of emotion regulation, a parent's behavior “scaffolds” a child's emotion regulation development if it is tailored to the developmental level and degree of needs of the child in a particular context, faded as the child begins to demonstrate more competence in handling the context, and is done in a way that the child gains more understanding and responsibility for emotion regulation. For instance, adaptive situation selection for children involves the developmentally appropriate provision of guidance from parents, who need to assist in choosing the appropriate contexts (e.g., children may not want to go to the dentist, but parents rightly override that situation selection choice). As well, through shared attention and engagement in emotion-eliciting situations, parents help their children to attend adaptively to modulate their distress (Raver, 1996).

Parental emotional scaffolding has been associated with emotion regulation in children with and without ASD, although the research is still sparse. Mothers of children with and without ASD are able to synchronize their behaviors to their children's attention and behavior (Siller & Sigman, 2002; Watson, 1998), and mothers of children with ASD are able to adapt their scaffolding to their children's individual levels of need, both of which are critical for successful scaffolding (Kasari, Sigman, Mundy, & Yimiya, 1988; Konstantareas, Zajdeman, Homatidis, & McCabe, 1988). Parents are often integral parts of behavioral intervention programs for children with ASD, and there is substantial evidence that providing parents with the skills to implement pivotal response training (a form of applied behavior analysis) can assist in teaching children with ASD self-management skills (Koegel & Kern Koegel, 2006; Schreibman & Koegel, 1996). When scaffolding is present, children with developmental disabilities show greater improvements in social skills, including aspects of emotional regulation (Baker, Fenning, Crnic, Baker, & Blacher, 2007), and more recently, Gulsrud et al. (2010) demonstrated that children with ASD and their parents “co-regulate” emotions during distressing events. In this latter study, 34 mother–child dyads participated in a joint attention intervention, and the authors studied children's emotion regulation through behavioral observation across all intervention sessions. The authors found that mothers (and children) were more likely to use emotion regulation strategies during periods of distress compared to periods without distress and that child comfort strategies (physical self-comfort and comfort seeking) were correlated with maternal active (prompting/helping, redirection of attention, and providing physical comfort) and vocal strategies (vocal comfort and reassurance). Of interest, maternal scaffolding was positively related to children's levels of externalizing behaviors, suggesting that mothers are more likely to employ scaffolding in the presence of challenging behavior. Perhaps most important, over the course of the 24-session joint engagement intervention, mothers demonstrated more emotional scaffolding, commensurate with children demonstrating improved emotional regulation.

Recent advances in the treatment of anxiety disorders in youth with ASD underscore how parents play a role in the development and treatment of their children's anxiety disorders (Reaven, 2011; Wood et al., 2009). Reaven and Hepburn (2006; see also Reaven, 2011) describe the critical role parents play in the maintenance of anxiety in youth with ASD, and these influences can be applied to emotion regulation more broadly. Parents of youth with ASD are more likely to experience parenting stress and parental anxiety compared to parents of typically developing youth, and such states may predispose parents to tolerate children's maladaptive emotion regulation strategies (e.g., avoidance), preventing the development of adaptive coping. In turn, the failure to develop a repertoire of adaptive strategies leads to less perceived competence and the perpetuation of maladaptive strategies in challenging situations. Parent-involved cognitive behavior therapy leads to greater changes in anxiety for children with ASD compared to child-only CBT or to waitlist controls (Puleo & Kendall, 2011; Sofronoff et al., 2005), lending support to the need to involve parents in children's emotion regulation treatment.

Peers and authority figures (i.e., teachers) also play critical roles in influencing the growth and implementation of emotion regulation (Jahromi, Bryce, & Swanson, 2013). In typically developing children, self-regulation is correlated with emotional and behavioral engagement in school (Denham et al., 2003; Valiente, Lemery-Chalfant, & Castro, 2007; Valiente, Lemery-Chalfant, Swanson, & Reiser, 2008) and with prosocial peer engagement and peer acceptance (Valiente, Swanson, & Lemery-Chalfant, 2012). More recently, emotional regulation has been correlated with prosocial peer engagement in preschool children with ASD (Jahromi et al., 2013). Unfortunately, individuals with ASD are more likely to have relationships with atypical social agents in their environment (e.g., educational assistants, therapists, and other specialized support staff; Bauminger & Kasari, 2000; Daniel & Billingsley, 2010; Humphrey & Symes, 2010; Mesibov & Shea, 1996) as well as negative typical relationships (e.g., peers and teachers), and these relationships can have a profound impact on the cognitive schemata children form about themselves, others, and the future, greatly impacting their social-emotional characteristics (Bauminger, 2007). It is important to consider the various ways that peers can be involved in supporting children's development in the five discussed components of emotion regulation. For instance, there is emerging evidence that peer supports (such as peer “buddies”) can be used as a part of cognitive-behavioral interventions for anxiety in youth with ASD to provide ecologically valid opportunities for social and emotional development (White et al., 2013; Wood et al., 2009). Typically developing peers have also been used to teach complex social behaviors to children with ASD through pivotal response training (Pierce & Schreibman, 2013), highlighting how peers may be instrumental in teaching individuals with ASD complex socioemotional skills, including those involved in emotion regulation.

## Conclusion

A disorder-specific approach to treating emotional problems may miss the main underlying maladaptive processes that can lead to problems with anxiety, depression, or anger, which are often found to be co-occurring in youth with ASD. A transdiagnostic framework has been effectively employed to treat emotion regulation impairments in typically developing youth with anxiety and depression (Ehrenreich-May & Bilek, 2012), and the components of this model have direct relevance to maladaptive processes often found in youth with ASD. Assessing maladaptive situation selection, situation modification, attentional deployment, cognitive change, and response modulation in youth with ASD may prove to be a useful method of formulating individual treatment plans to address emotional problems at a transdiagnostic level. Further research is needed to empirically test how a transdiagnostic intervention can address the processes of regulation and assist youth with ASD to cope with anger, anxiety, and depression.

## Notes

1 A more recent pilot study of a smaller sample of adolescents with high-functioning ASD, using a newly modified psychiatric interview, suggests that earlier reports of rates may be overestimated by not comprehensively taking into account ASD symptoms, although even here rates continue to remain high (Mazefsky, Oswald, et al., 2012).
